# Biosynthesis of ergothioneine: current state, achievements, and perspectives

**DOI:** 10.1007/s00253-025-13476-4

**Published:** 2025-04-12

**Authors:** Shun Sato, Azusa Saika, Tatsuyuki Koshiyama, Yukihiro Higashiyama, Tokuma Fukuoka, Tomotake Morita

**Affiliations:** 1 Research Institute for Sustainable Chemistry, National Institute of Advanced Industrial Science and Technology (AIST), Central 5, 1-1-1 Higashi, Tsukuba, Ibaraki 305-8565 Japan; 2 New Business Division, Kureha Corporation, 3-3-2 Nihonbashi-Hamacho, Chuo-ku, Tokyo, 103-8552 Japan

**Keywords:** Ergothioneine, Antioxidant, Basidiomycetes, Biosynthesis

## Abstract

**Abstract:**

Ergothioneine (EGT) is a derivative of the amino acid L-histidine that is well known for its strong antioxidant properties. Recent studies on the functional characterization of EGT in both in vivo and in vitro systems have demonstrated its potential applications in pharmaceuticals, food, and cosmetics. The growing demand for EGT in novel applications necessitates the development of safe and cost-effective mass production technologies. Consequently, microbial fermentation for EGT biosynthesis has attracted significant attention. This review focuses on the biosynthesis of EGT via microbial fermentation, explores its biosynthetic mechanisms, and summarizes the latest advancements for industrial EGT production using engineered microbial strains.

**Key points:**

• *Ergothioneine (EGT) is an L-histidine derivative with strong antioxidant property.*

• *Recent studies have revealed certain groups of microbes produce EGT naturally.*

• *Superior EGT producers by genetic modification have been created.*

## Introduction

Sulfur-containing biomolecules such as biotin, thiamine, and coenzyme A are essential to living organisms due to their critical roles in energy metabolism as cofactors in enzymatic reactions. Another sulfur-containing biomolecule, the tripeptide glutathione, is an antioxidant that protects cells from oxidative damage. In human cells, some sulfur-containing biomolecules are synthesized from sulfur-containing amino acids such as cysteine or methionine. However, others cannot be synthesized by the human body despite their essentiality, necessitating dietary intake. Deficiencies in these compounds often result in various diseases and symptoms (Smith et al. 2021; Yang et al. 2023). Consequently, medicines and dietary supplements comprising sulfur-containing biomolecules are widely distributed to aid in disease management and improve quality of life.

**Fig. 1:**
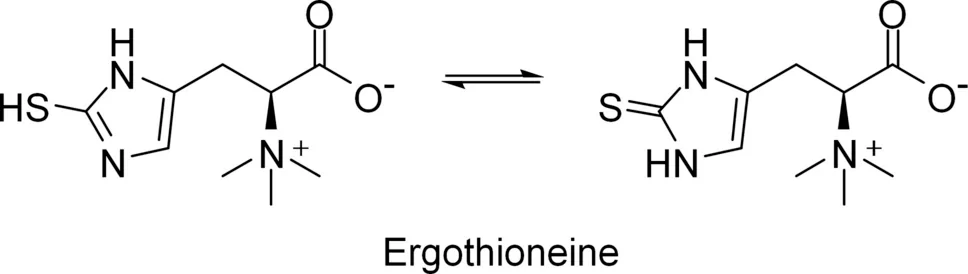
Chemical structure of EGT. The tautomer forms of thiol (left) and thione (right) are shown

Ergothioneine (EGT) is a sulfur-containing biomolecule derived from L-histidine that was first identified in the ergot fungus *Claviceps purpurea* (Tanret 1909). Its chemical structure involves a betaine configuration with a sulfur atom attached to the imidazole ring, and thiol and thione forms exist as a tautomer (Fig. 1). The thione form dominates at physiological pH (Servillo et al. 2015), making EGT stable in spite of the presence of the thiol group. EGT has since been detected in various human and animal cells and organs, including the liver, kidney, semen, and red blood cells (Cheah and Halliwell 2012; Borodina et al. 2020). Several studies have demonstrated the benefits of EGT (Colognato et al. 2006; Yang et al. 2012; Servillo et al. 2015; D'Onofrio et al. 2016; Smith et al. 2020), positioning it as a vitamin-like compound for humans (Paul and Snyder 2010). However, as plants and animals, including humans, cannot synthesize EGT, its presence in these organisms is believed to originate from microorganisms in natural or symbiotic environments. Mushrooms are recognized as the richest natural source of EGT (Kalaras et al. 2017), and mushroom extracts containing EGT are commercially available (Fu and Shen 2022).

Recent studies have proposed novel applications for EGT in various industries such as foods, cosmetics, and agriculture. Encarnacion et al. (2011) demonstrated prevention of postharvest melanosis of shrimp by treating it with mushroom extracts containing EGT. Hseu et al. (2020) reported the protective effect of EGT on ultraviolet-induced damaged human dermal fibroblasts via controlling expression of genes concerning extracellular matrix-degradation and antioxidant. Hanayama et al. (2024) found skin moisturizing functions being improved by oral intake of EGT-containing mushroom products. Zhang et al. (2023b) reported preventing effect of EGT treatment on *Brassica rapa* clubroot development via elevating transcription levels of biosynthetic genes for phenolic compounds. Koshiyama et al. (2024) revealed that seed productivity of *Arabidopsis thaliana* increased by EGT via elevating transcript levels of *FLOWERING LOCUS T*. Sivakumar and Bozzo (2024) demonstrated preservative effect on postharvest arugula by EGT treatment. More recent studies suggested lifespan extending effect of EGT on *Drosphila melanogaster* (Pan et al. 2022, 2024). These advancements have significantly increased the demand for EGT. However, conventional manufacturing processes involving mushroom cultivation, fruiting body harvesting, and extraction are time-consuming and inefficient. Meeting the growing demand for EGT requires a safe, cost-effective process for its mass production.

This review focuses on the biosynthesis of EGT through microbial fermentation and its underlying biosynthetic mechanisms. Additionally, it summarizes recent technological advancements for the industrial production of EGT using engineered microbial strains.

## EGT in microbial cells

The first identification of EGT in microbial cells was reported in the ergot fungus *C. purpurea*, a member of phylum Ascomycota (Tanret 1909). Subsequent studies have confirmed the presence of EGT in various bacteria and fungi, including mushrooms (Table 1).

**Table 1:** Microorganisms identified as native EGT producers

Phylum	Class	Genus	Species	References
Fungi				
Ascomycota	Dothideomycetes	*Alternaria*	*zinniae*	Melville et al. (1956)
	Eurotiomycetes	*Aspergillus*	*carbonarius*, *fumigatus*, *nidulans*, *niger*, *oryzae*, *tenuis*	Melville et al. (1956); Genghof (1970); Gallagher et al. (2012); Takusagawa et al. (2019)
		*Penicillium*	*notatum*, *roqueforti*	Genghof (1970)
	Dipodascomycetes	*Geotrichum*	*rugosum*	Genghof (1970)
	Dothideomycetes	*Aureobasidium*	*pullulans*	Melville et al. (1956); Fujitani et al. (2018)
	Pezizomycetes	*Morchella*	*esculenta* ^a)^	Kalaras et al. (2017)
	Schizosaccharomycetes	*Schizosaccharomyces*	*pombe*	Pluskal et al. (2010)
	Sordariomycetes	*Claviceps*	*purprea*	Tanret (1909)
		*Cordyceps*	*militaris* ^a)^	Chan et al. (2015)
		*Neurospora*	*crassa*, *tetrasperma*	Genghof (1970)
Basidiomycota	Agaricomycetes	*Agaricus*	*bisporus* ^a)^	Kalaras et al. (2017)
		*Agrocybe*	*aegerita* ^a)^	Kalaras et al. (2017)
		*Boletus*	*edulis* ^a)^	Kalaras et al. (2017)
		*Cantharellus*	*cibarius* ^a)^	Kalaras et al. (2017)
		*Flmmulina*	*velutipes* ^a)^	Bao et al. (2008)
		*Ganoderma*	*lucidum* ^a)^, *resinaceum* ^a)^	Kalaras et al. (2017); Yu et al. (2025)
		*Grifola*	*frondose* ^a)^	Kalaras et al. (2017)
		*Hericium*	*erinaceus* ^a)^	Kalaras et al. (2017)
		*Lentinula*	*edodes* ^a)^	Tepwong et al. (2012); Kalaras et al. (2017)
		*Lyophyllum*	*connatum* ^a)^	Kimura et al. (2005)
		*Pleurotus*	*citrinpileatus* ^a)^, *cornucopiae* ^a)^, *eryngii* ^a)^, *ostreatus* ^a)^	Liang et al. (2013); Lin et al. (2015); Kalaras et al. (2017); Fujitani et al. (2018)
		*Panus*	*conchatus* ^a)^	Zhu et al. (2022)
	Microbotryomycetes	*Rhodotorula*	*glutinis, mucilaginosa*	Fujitani et al. (2018)
		*Sporobolomyces*	*salmonicolor*	Genghof (1970)
	Ustilaginomycetes	*Anthracocystis*	*flocculosa*	Sato et al. (2024)
		*Dirkmeia*	*churashimaensis*	Sato et al. (2024)
		*Kalmanozyma*	*fusiformata*	Sato et al. (2024)
		*Moesziomyces*	*antarcticus*, *parantarcticus*, *rugulosus*	Sato et al. (2024)
		*Pseudozyma*	*alboarmeniaca*, *graminicola*, *hubeiensis*, *prolifica*,* tsukubaensis*	Fujitani et al. (2018); Sato et al. (2024)
		*Triodiomyces*	*crassus*	Sato et al. (2024)
		*Ustilago*	*maydis*, *shanxiensis*, *siamensis*	Sato et al. (2024)
Mucoromycota	Mucoromycetes	*Mucor*	*mucedo*	Melville et al. (1956)
		*Rhizopus*	*stolonifer*	Genghof (1970)
Bacteria				
Actinomycetota	Actinomycetes	*Actinoplanes*	*philippinensis*	Genghof (1970)
		*Mycobacterium*	*avium*, *bovis*, *fortuitum*, *intracellularis*, *kansasii*, *leprae*, *marinum*, *microti*, *neoaurum*, *paratubeculosis*, *phlei*, *piscium*, *rhodochrous*, *smegmatis*, *thamnopheos*, *tubeculosis*, *ulcerans*	Genghof and van Damme (1964); Xiong et al. (2022)
		*Nocardia*	*asteroides*	Genghof (1970)
		*Streptomyces*	*albus*, *coelicolor*, *fradiae*,* griseus*	Genghof (1970); Nakajima et al. (2015)
Cyanobacteriota	Cyanophyceae	*Oscillatoria*	sp.	Pfeiffer et al. (2011)
		*Scytonema*	sp.	Pfeiffer et al. (2011)
Pseudomonadota	α-Proteobacteria	*Methylobacterium*	*aquaticum*	Alamgir et al. (2015)
Rhodothermota	Rhodothermia	*Salinibacter*	*ruber*	Burn et al. (2017)

^a)^Mushrooms

Currently, mushrooms are the most well-known natural producers of EGT. Phylum Ascomycota includes non-edible fungi, such as *Cordyceps militaris* (class *Sordariomycetes*), a medicinal fungus known for producing cordycepin, which also contains EGT in its fruiting bodies (Chan et al. 2015). The yellow morel *Morchella esculenta* (class Pezizomycetes) has also been identified as an EGT producer (Kalaras et al. 2017). Phylum Basidiomycota includes widely consumed edible mushrooms capable of producing EGT, such as *Agaricus bisporus* (white mushroom), *Boletus edulis* (porcini), and *Pleurotus citrinopileatus* (golden oyster mushroom), all belonging to class Agaricomycetes. Additionally, medicinal mushrooms, including *Ganoderma lucidum* and *Panus conchatus*, are known as EGT producers (Kalaras et al. 2017; Zhu et al. 2022). However, large-scale production of EGT through mushroom cultivation is inefficient due to the extended time required for fruiting body maturation (several weeks or months depending on the species) and the relatively low EGT content (0.15–7.27 mg/g dry mushrooms) in these organisms (Dubost et al. 2007; Pfeiffer et al. 2011; Kalaras et al. 2017). Therefore, extensive research has been conducted to identify microbial sources that can facilitate fermentative EGT production.

Among fungi other than mushrooms, several members of Ascomycota have been reported to produce EGT, including *Neurospora crassa* (class Sordariomycetes), *Aspergillus fumigatus* and *Penicillium notatum* (class Eurotiomycetes), and *Aureobasidium pullulans* (class Dothideomycetes) (Melville et al. 1956; Genghof 1970; Fujitani et al. 2018). In the fission yeast *Schizosaccharomyces pombe* (class Schizosaccharomycetes), the genetic and biochemical pathways for EGT biosynthesis have been extensively investigated (Pluskal et al. 2010, 2014). Many EGT-producing fungi belong to phylum Basidiomycota, and yeast species within this phylum have also been identified as EGT producers, such as strains of *Rhodotorula* and *Sporobolomyces* (class Microbotryomycetes) (Genghof 1970; Fujitani et al. 2018). Recent studies have identified diverse EGT-producing yeast strains within class Ustilaginomycetes (Sato et al. 2024), a group also known for glycolipid production (Morita et al. 2015). Phylum Mucoromycota includes EGT-producing genera such as *Mucor* and *Rhizopus* (Melville et al. 1956; Genghof 1970). However, some yeast strains within Ascomycota, including *Saccharomyces cerevisiae*, *Pichia membranifaciens*, *Candida albicans*, and *Torulopsis utilis*, have been reported to lack EGT production (Genghof 1970; Fujitani et al. 2018).

In bacteria, multiple strains of *Mycobacterium* (phylum Actinomycetota) have been identified as EGT producers. Other actinomycetes capable of EGT synthesis include *Actinoplanes philippinensis* and *Nocardia asteroides* (Genghof 1970). Certain *Streptomyces* strains, including *Streptomyces albus* and *Streptomyces griseus*, which are known for antibiotic production, also synthesize EGT (Genghof 1970). Other bacterial taxa, including cyanobacteria and *Methylobacterium*, have been identified as EGT producers (Pfeiffer et al. 2011; Alamgir et al. 2015). More recently, *Salinibacter ruber*, an extremely halophilic bacterium within phylum Rhodothermota, was demonstrated to produce EGT (Burn et al. 2017). The gene involving EGT biosynthesis under anaerobic conditions was found in this bacterium. Despite these findings, few EGT-producing bacterial species have been identified compared to fungi. Furthermore, several bacterial species have been reported to lack EGT production, including *Corynebacterium xerosis* and *Micrococcus pyogenes var. aureus* (phylum Actinomycetota); *Bacillus subtilis*, *Clostridium perfringens*, and *Lactobacillus casei* (phylum Bacillota); and *Escherichia coli*, *Pseudomonas fluorescens*, and *Vibrio metchnikovii* (phylum Pseudomonadota) (Melville et al. 1956).

## EGT synthetic reactions

**Scheme 1:**
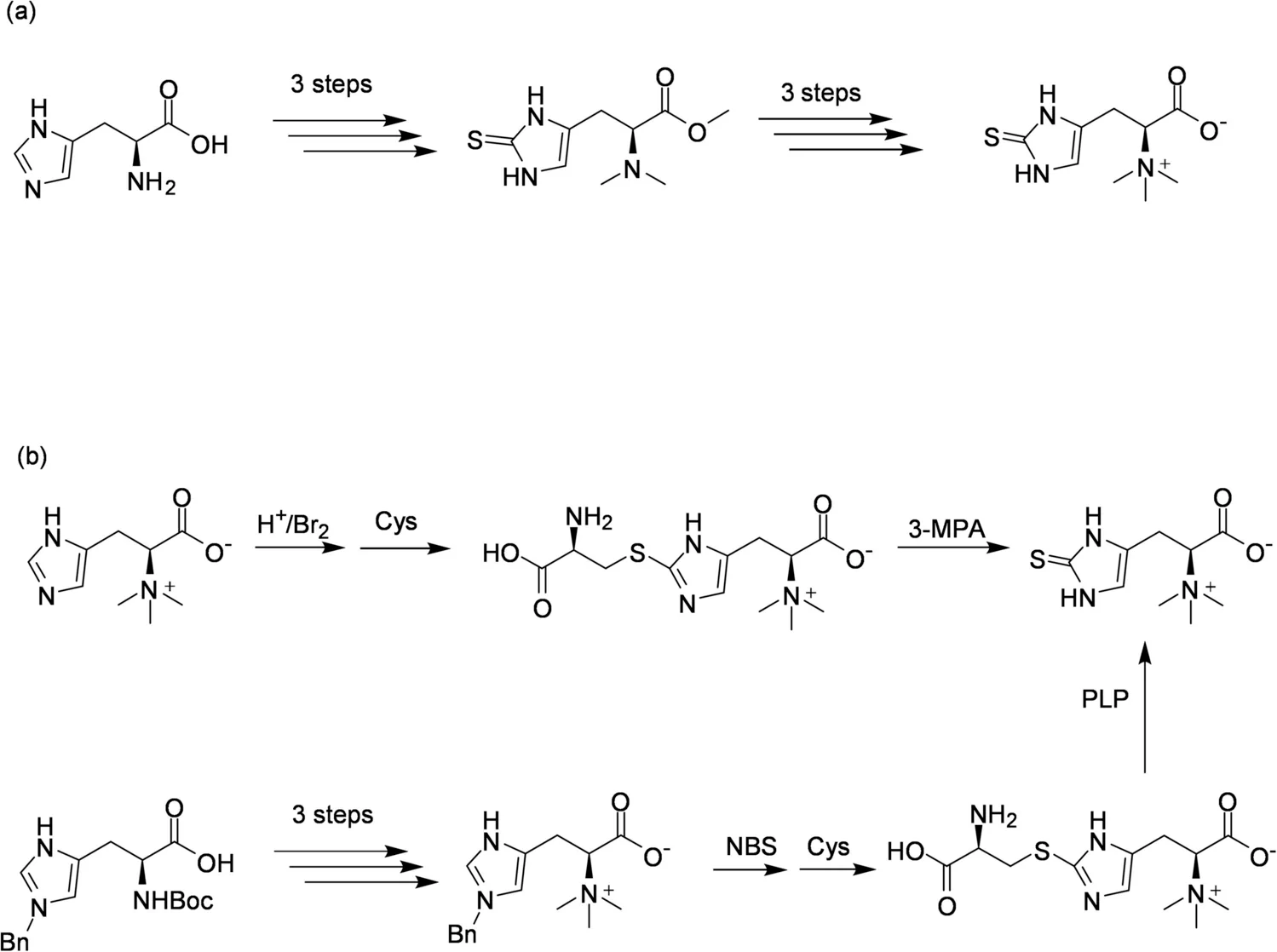
Cys, L-cysteine; 3-MPA, 3-mercaptopropionic acid; NBS, *N*-bromosuccinimide; PLP, pyridoxal phosphate

EGT can be synthesized from L-histidine and its derivatives through enzymatic reactions both in vivo and in vitro, as well as through chemical reactions. Although the chemical structure of EGT is relatively simple (Fig. 1), its chemical synthesis is complicated by the need for protection and deprotection of carboxyl and amino groups when introducing a sulfur atom at the C- 2 position of the imidazole ring. These reactions require harsh conditions, involve harmful chemicals, and result in low yields. Xu and Yadan (1995) reported the synthesis of EGT from L-histidine via an *N*α,*N*α-dimethyl imidazole- 2-thione derivative in six steps, with an overall yield of 34% (Scheme 1a). The key sulfur introduction reaction was achieved using phenyl chlorothionoformate as the sulfur donor. Recent studies have developed sulfur-introducing reactions that mimic biological processes in microbial cells for EGT synthesis under milder conditions with fewer reaction steps. Using hercynine as a starting material, EGT can be synthesized in a single-pot reaction via hercynylcysteine sulfide as an intermediate (Erdelmeier et al. 2012; Scheme 1b). Hercynylcysteine sulfide can also be synthesized from an L-histidine derivative with an *N*-benzyl-protected imidazole in good yield, followed by a non-enzymatic reaction with pyridoxal phosphate to yield EGT (Khonde and Jardine 2015; Scheme 1b).

**Fig. 2:**
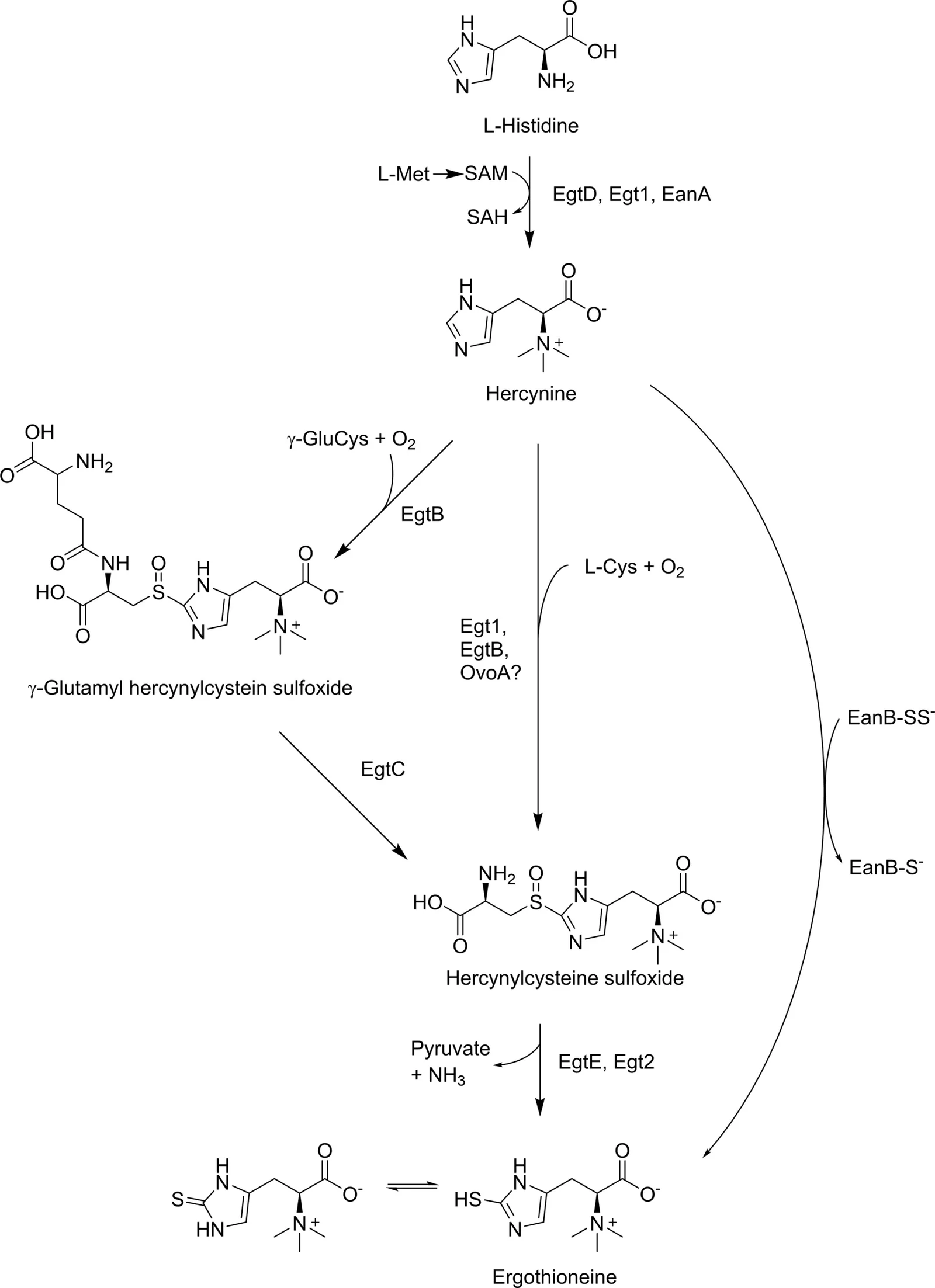
Biosynthetic pathways for EGT production in *Mycobacterium smegmatis* (left), *Neurospora crassa* (center), and *Chlorobium limicola* (right). Met, L-methionine; SAM, *S*-adenosylmethionine; SAH, *S*-adenosylhomocysteine; Cys, L-cysteine; γ-GluCys, γ-glutamylcysteine

The synthesis of EGT in microbial cells is relatively simple and efficient. The synthetic routes in microbial cells have been investigated, and the catalytic enzymes involved have been identified. In EGT-producing bacteria such as *Mycobacterium smegmatis*, EGT is synthesized from L-histidine via four enzymatic reactions (Fig. 2, left). *N*α-trimethylation of L-histidine with three molecules of *S*-adenosylmethionine is catalyzed by EgtD, followed by sulfur introduction into the imidazole ring, catalyzed by EgtB, using γ-glutamylcysteine as a sulfur donor. EgtC catalyzes the cleavage of glutamic acid, followed by C-S cleavage to produce ergothioneine with EgtE. In addition to these four enzymes, EgtA, which catalyzes the formation of γ-glutamylcysteine from glutamic acid and cysteine, has been identified. These five enzyme-encoding genes form a cluster for EGT biosynthesis.

In EGT-producing fungi, including *N. crassa*, EGT is synthesized via three enzymatic reactions, which is more efficient than the bacterial pathway (Fig. 2, center). First, L-histidine is converted to hercynine by Egt1 using SAM. The same protein, with an additional enzymatic activity, then catalyzes the introduction of cysteine at the C- 2 position of the imidazole ring in hercynine, forming cysteinyl hercynylsulfoxide. This step differs from the bacterial pathway, which uses γ-glutamylcysteine. The subsequent reaction forming EGT is catalyzed by Egt2. As for *Flammulina velutipes*, an edible mushroom known as the EGT producer (see Table 1), three genes encoding enzymes for EGT biosynthesis were identified and in vitro formation of EGT using the three enzymes (FvEgt1, 2, and 3) was demonstrated (Yang et al. 2020). In the reaction, formation of cysteine hercynylsulfoxide from L-histidine is catalyzed by FvEgt1, as the same as that in other fungi, while liberation of EGT from cysteinyl hercynylsulfoxide is catalyzed by FvEgt2 and FvEgt3 simultaneously. Another mushroom *Grifola frondosa* was found to possess EGT biosynthetic genes *egt1* and *egt2*, both of which only required EGT biosynthesis in *S. cerevisiae* (Yu et al. 2020).

EgtB, the key enzyme in EGT biosynthetic reactions, has been categorized into five types based on structural perspectives (Stampfli et al. 2019). Type II EgtB from *Chloracidbacterium thermophilum* exhibited catalytic activity toward cysteine rather than γ-glutamylcysteine in in vitro enzymatic characterization. Some EgtBs from *Methylobacterium* strains, classified as type II, have also been demonstrated to produce EGT by heterologous expression of *egtB* genes in *E. coli* without EgtC activity (Kamide et al. 2020).

Recently, additional biosynthetic routes have been discovered. The strictly anaerobic photosynthetic green sulfur bacterium *Chlorobium limicola* possesses EanA and EanB enzymes, which catalyze the anaerobic biosynthesis of EGT from histidine (Burn et al. 2017). In the EanB-catalyzing reaction, sulfur is introduced into the imidazole ring of hercynine without sulfur donor molecules such as cysteine, occurring in an oxygen-independent manner that differs from other EGT biosynthetic pathways (Fig. 2, right). The sulfur donor in this reaction is EanB itself with the catalytic cysteine persulfide anion (EanB-SS^−^ in Fig. 2), which attacks the imidazole ring of hercynine directly (Leisinger et al. 2019). Genes with high sequence identity to *eanA* and *eanB* have been discovered in other bacteria and archaea, including *S. ruber* and *Methanohalophilus mahii* (Burn et al. 2017). In cyanobacteria, sulfoxide synthase activity involved in EGT biosynthesis may be provided by OvoA, an enzyme involved in the biosynthesis of ovothiol A, another thiol-containing L-histidine derivative (Liao and Seebeck 2017). These discoveries provided insight into the biological functions of EGT in EGT-producing microbes, as well as alternative tools for synthetic biology to create superior EGT producers.

## Fermentative production of EGT by native EGT producers

Identified native EGT producers have been investigated for their fermentative production of EGT, as summarized in Table 2. In fungi, early studies reported EGT production at levels below 1 mg/g dry cells, including species such as *Aspergillus niger*, *N. crassa*, and *P. notatum* (Melville et al. 1956). More recent studies on novel EGT producers have demonstrated fermentative production with productivity exceeding 1 mg/g dry cells or 10 mg/L culture medium. Fujitani et al. (2018) investigated *A. pullulans* and *Rhodotorula mucilaginosa* for EGT production and, after optimizing culture conditions, achieved fermentative production levels of 14 and 24 mg/L, respectively.

**Table 2:** Biosynthesis of EGT by native EGT-producing microbes

Organisms	Culture media	Scales	Titer	Specific yield	Productivity	References
			(mg/L culture)	(mg/g dry cell)	(mg/L/day)	
Fungi						
*Aspergillus niger* ATCC1027	Czapek’s solution	Flask	-	0.22	-	Melville et al. (1956)
*Aspergillus oryzae* NSAR1	Steeped rice solid medium		11.5 (mg/kg-media)	-	2.3	Takusagawa et al. (2019)
*Alternaria zinniae* ATCC11786	Czapek’s solution	Flask	-	0.02	-	Melville et al. (1956)
*Aureobasidium pullulans* kz25	SD medium, 2% glycerol + 2% yeast extract	Test tube	14	1	2	Fujitani et al. (2018)
*Mucor mucedo* ATCCC9836	Czapek’s solution	Flask	-	0.29	-	Melville et al. (1956)
*Neurospora crassa* ATCC10337	Ryan	Flask	-	0.86	-	Melville et al. (1956)
*Panus conchatus* (mycelia)	Optimized fermentation medium, 5% molasses, 3% soypeptone	Flask	81.44	8.4	20.4	Zhu et al. (2022)
	Optimized fermentation medium, 5% molasses, 3% soypeptone, 0.04% cysteine	Flask	148.79	9.1	24.8	Zhu et al. (2022)
*Penicillium notatum* ATCC8537	Wickerham medium	Flask	-	0.13	-	Melville et al. (1956)
*Pleurotus citrinopileatus* (mycelia)	Basal medium, 2% glucose		18.2	2.9	0.83	Lin et al. (2015)
	Basal medium, 2% glucose + amino acids		98	12.3	6.1	Lin et al. (2015)
*Rhodotorula mucilaginosa* z41c	SD medium, 2% glycerol + 2% yeast extract	Test tube	24	3.2	3.4	Fujitani et al. (2018)
*Shizossacharomyces pombe* WT 972	EMM2 medium, nitrogen starvation		-	157.4 (μM, intracellular)	-	Pluskal et al. (2014)
*Tridiomyces crassus* CBS9959	YM medium (flask)	Flask	30.9 ± 1.8	5.7	6.2	Sato et al. (2024)
*Ustilago shanxiensis* CBS10075	YM medium, 0.1% methionine (flask)	Flask	34.7 ± 3.9	8.4	6.9	Sato et al. (2024)
*Ustilago siamensis* CBS9960	YM medium (flask)	Flask	49.5 ± 7.0	9.3	9.9	Sato et al. (2024)
	YM medium (jar)	5L-Jar	54.0 ± 15.0	11.7	10.8	Sato et al. (2024)
	YM medium, 0.1% histidine (flask)	Flask	74.9 ± 5.5	13.9	15	Sato et al. (2024)
Bacteria						
*Methylobacterium aquaticum* 22 A	MM medium, 2% methanol	Flask	12.2	2	1.7	Fujitani et al. (2018)
*Mycolicibacterium neoaurum* ATCC25795	Chemically defined medium containing 1.6% glycerol, 0.4% glucose, 0.2% citric acid	Flask	13.3	-	2.66	Xiong et al. (2022)
*Nocardia asteroids*	Wickerham medium, 1% mannitol + 0.4% asparagine		-	0.52	-	Genghof (1970)
*Oscillatoria* sp. CCAC M1944	Waris-H medium	Flask	-	0.8–0.9	-	Pfeiffer et al. (2011)
*Streptomyces griseus* ATCC10317	Romano and Nickerson medium		-	0.5	-	Genghof (1970)

Among the Basidiomycota, diverse yeast strains belonging to basidiomycetes have been identified as EGT producers (Sato et al. 2024). *Ustilago siamensis* produces EGT at 49.5 mg/L in yeast malt medium and 74.9 mg/L in yeast malt medium supplemented with histidine. The intracellular EGT content in *U. siamensis* reached 13.9 mg/g dry cells, representing the highest productivity among native EGT producers. This strain was also capable of EGT production in a jar fermenter, indicating its potential for large-scale production.

Additionally, submerged mycelial cultivation of mushrooms has been demonstrated to efficiently produce EGT. After optimizing culture conditions, EGT production in the golden oyster mushroom *P. citrinopileatus* improved to 98 mg/L under mycelium cultivation (Lin et al. 2015). Another mushroom, *P. conchatus*, was also cultivated for EGT production, achieving 149 mg/L within 6 days (Zhu et al. 2022), corresponding to a high productivity rate of 24.8 mg/L/day. These findings suggest the potential for large-scale EGT production through mycelial culture of EGT-producing mushrooms.

In addition to fungal sources, bacterial strains of native EGT producers have been studied for their EGT production. Strains of cyanobacterial genera including *Scytonema* and *Oscillatoria* produced intracellular EGT at levels of up to 0.8 mg/g dry cells, which was higher than the levels found in king oyster mushrooms (Pfeiffer et al. 2011). An investigation of EGT production in *Methylobacterium aquaticum* 22 A revealed EGT accumulation of 12.2 mg/L (Fujitani et al. 2018). *Mycolicibacterium neoaurum* produced EGT at 13.3 mg/L in a chemically defined medium containing glycerol, glucose, and citric acid as carbon sources (Xiong et al. 2022).

## Genetically modified microorganisms for EGT production

Following the identification of EGT biosynthetic genes in *M. smegmatis* (Seebeck 2010), *N. crassa* (Bello et al. 2012), *S. pombe* (Pluskal et al. 2014), and *F. velutipes* (Yang et al. 2020), EGT-producing microbes have been created using genetic engineering technology, as shown in Table 3.

**Table 3:** Biosynthesis of EGT in genetically modified microbes

Organisms	Strain	Parental strain	Genes modified ^a)^	Conditions	Titer	Specific yield	Productivity	References
					(mg/L culture)	(mg/g dry cell)	(mg/L/day)	
Fungi								
*Aspergilus oryzae*	NS-Nc12	NSAR1	*egt1* and *egt2* from *Neurospora crassa*	Solid media, 120 h	231.0 ± 1.1 mg/kg of media	-	46.2 mg/kg/day	Takusagawa et al. (2019)
*Cordyceps militaris*	15-E1bD2	CM15	*egtD from Mycobacterium smegmatis, truncated egt1 and egt2*	Flask, 240 h	-	2.49 ± 0.05	-	Chen et al. (2022)
*Saccharomyces cerevisiae*	ST8927	CEN.PK113 - 7D	*egt1* from *Neurospora crassa* and *egt2* from *Claviceps purpurea*; 2 copies	1-L jar, fed-batch, 84 h	598 ± 18	-	171	van der Hoek et al. (2019)
*Saccharomyces cerevisiae*	ST10165	ST8927 (egt1 and egt2 inserted strain of CEN.PK113 - 7D)	His-overproducing mutant, *met14*, Δ*spe2*	1-L jar, fed-batch, 160 h	2390 ± 80	-	359	van der Hoek et al. (2022a)
*Saccharomyces cerevisiae*	BY4741^egtA^	BY4741	*egtA* from *Aspergillus fumigastus*	Flask, 96 h	7.93	-	-	Doyle et al. (2022)
*Saccharomyces cerevisiae*	IMX582-Egt1&2-STL1	IMX581	*egt1 from Ganoderma resinaceum and egt2 from Neurospora crassa, STL1-overproducing*	5-L jar, fed-batch, 240 h	1140	-	114	Yu et al. (2024)
*Schizosaccharomyces pombe*	972	*P3nmt-egt1*^+^	*egt1* from *S. pombe*	Synthetic minimal medium (EMM2)	-	1.6 mM	-	Pluskal et al. (2014)
*Yarrowia lipolytica*	ST10264	ST6512	*egt1* from *Neurospora crassa* and *egt2* from *Claviceps purpurea*; 2 copies	1-L jar, fed-batch, 220 h	1637 ± 41	-	178	van der Hoek et al. (2022b)
Bacteria								
*Crynebacterium glutamicum*	ET11	ATCC13032	*egt1* and *egt2* from *Schizosaccharomyces pombe*, *cysEKR* from *C. glutamicum*, promoter replacement by H36 synthetic promoter for enhancing sulfur assimilation, pentose phosphate pathway and cysteine synthesis, Δ*sdaA*	Jar, fed-batch, 36 h	264.4	-	176	Kim et al. (2022)
*Crynebacterium glutamicum*	CYS- 2/pECt-Mb_egtB-Ms_egtDE	CYS- 2 (cystein-overproducing strain)	*egtB* from *Methylobacterium brachiatum*, *egtDE* from *Mycolicibacterium smegmatis*	Flask, fed-batch, 120 h	100	-	20	Hirasawa et al. (2023)
*Escherichia coli*	ET3	BW25113 (high L-cysteine producing strain)	*egtBCDE* from *Mycobacterium smegmatis*	Flask, 72 h	24 ± 4	-	8	Osawa et al. (2018)
*Escherichia coli*	CHΔmetJ pQE1a-egtABCDE	JW3909 (*metJ*-deleted strain of BW25113)	*egtABCDE* from *Mycobacterium smegmatis*, Δ*metJ*	3-L jar, fed-batch, 216 h	1310	-	146	Tanaka et al. (2019)
*Escherichia coli*	BW-tregt1-tregt2	BW25113	*egt1* and *egt2* from *Trichoderma reesei*	2-L jar, fed-batch, precursors supplemented, 143 h	4340	-	728	Chen et al. (2022)
*Escherichia coli*	MD4	BL21(DE3)	truncated, mutated *egt1* from *Neurospora crassa*, mutated *egtD* from *Mycrobacterium smegmatis*	Jar, fed-batch, precursors supplemented, 96 h	5400	-	1351	Zhang et al. (2023a)
*Escherichia coli*	ECE14	BL21(DE3)	*egtBDE* from *Methylobacterium aquaticum*, *serA*^mut^, *thrA*^mut^, Δ*metJ,*Δ*sdaA*,	10-L jar, fed-batch, 72 h	595	-	197	Zhang et al. (2024)
*Methylobacterium aquaticum*	22 AΔhutH(EGT)	22 A	*egtBD*, Δ*hutH*	Test tube, 168 h	20	7	2.9	Fujitani et al. (2018)
*Mycolicibacterium neoaurum*	EGT24E	ATCC25795	*egtABCDE*, *hisG*, *hisC*, *allB1*, deletion of putative EGTase gene (MnΔ3042)	5-L jar, fed-batch, precursorus supplemented, 216 h	1560 ± 270	-	173	Xiong et al. (2022)

^a)^Abbreviations: *egtA*, glutamine-cysteine ligase gene; *egtB*, hercynine oxygenase gene; *egtC*, glutamine amidotransamidase gene; *egtD*, *S*-adenosylmethionine-dependent methyltransferase gene; *egtE*, pyridoxal 5-phosphate-dependent b-lyase gene; *egt1*, bifunctional enzyme gene of *S*-adenosylmethionine-dependent methyltransferase and hercynine-cysteine sulfoxidase; *egt2*, pyridoxal 5-phosphate-dependent b-lyase gene; *metJ*, transcriptional repressor gene for methionine biosynthetic genes; *met14*, ATP: adenylylsulfate- 3′-phosphotransferase gene; *spe2*, *S*-adenosylmethionine decarboxylase gene; *hutH*, histidine-ammonia lyase gene; *hisG*, ATP-phosphoribosyltransferase gene; *hisC*, histidinol-phosphate aminotransferase gene; *allB1*, allantoinase 1 gene; *sdaA*, serine deaminase gene; *cysE*, serine acetyltransferase gene; *cysK*, *O*-acetyl L-serine sulfhydrylase; *cysR*, transcriptional regulator gene for sulfur assimilation

In fungi, non-EGT producers, such as *S. cerevisiae* and *Yarrowia lipolytica*, have been engineered to enable EGT production by heterologous expression of EGT biosynthetic genes from *N. crassa* and *C. purpurea*. By tuning the metabolic balance in precursor supply through gene manipulation and cultivation conditions, *S. cerevisiae* ST10165 and *Y. lipolytica* ST10264, both of which harbor two copies of *egt1* from *N. crassa* and two copies of *egt2* from *C. purpurea*, produced EGT at 2390 ± 80 and 1637 ± 41 mg/L from glucose after 160 and 220 h of fed-batch cultivation, respectively, using synthetic media without supplementation of precursor amino acids (van der Hoek et al. 2022a, 2022b). The strain *S. cerevisiae* ST10165 was genetically modified in L-histidine biosynthesis by mutagenesis with β-(1,2,4-triazol- 3-yl)-DL-alanine, SAM metabolism by *spe2* deletion, and L-cysteine fluxes by overexpression of *met14*, suggesting that enhancement of Egt1-catalyzing reaction (Fig. 2, center) could improve EGT productivity. In contrast, the strain *Y. lipolytica* ST10264 had no genetic modification for amino acid metabolisms and the cells were cultivated under phosphate-limited conditions, suggesting sufficient pools of precursor amino acids available for EGT biosynthesis in *Y. lipolytica*. Another strain of *S. cerevisiae*, harboring *egt1* from *Ganoderma resinaceum* and *egt2* from *N. crassa* for EGT synthesis and overexpressing *SLT1* for enhanced glycerol assimilation, produced EGT at 1.14 g/L from glycerol after 240 h of fed-batch cultivation using semisynthetic medium containing 10 g/L yeast extract and 20 g/L peptone as the starting medium (Yu et al. 2024). Interestingly, recombinant *S. cerevisiae* with *egtA*, which encodes methyltransferase and sulfoxide synthase from *A. fumigatus*, produced a small amount of EGT at 7.93 mg/L in flask cultivation (Doyle et al. 2022). Because no EGT biosynthetic genes are found in *S. cerevisiae*, the reaction catalyzed by Egt2 or EgtE, which involves C-S bond cleavage to form EGT, may have occurred due to inherent activity in *S. cerevisiae* or as an abiotic reaction.

Among bacterial hosts, *Escherichia coli*, a non-EGT producer, has been primarily used to confer EGT-producing ability through genetic engineering. Using *egtA*, *egtB*, *egtC*, *egtD*, and *egtE* from *M. smegmatis*, an *E. coli* cysteine-hyperproducing mutant acquired the ability to produce EGT. By modifying precursor supplies, *E. coli* strain CH*ΔmetJ* pQE1a-egtABCDE produced EGT at 1.3 g/L in 216 h of fed-batch cultivation with supplementation of L-histidine, L-methionine, and sodium thiosulfate after induction of expression of introduced genes (Tanaka et al. 2019). Deletion of *metJ*, the transcriptional repressor for L-methionine and SAM biosynthesis, in this strain could enhance SAM supply to EGT biosynthesis by elevating transcriptional level of *metK* that encodes methionine adenosyltransferase. Additionally, *E. coli* strains harboring *egt1* and *egt2* from the fungus *Trichoderma reesei* achieved EGT production of 4.34 g/L in 143 h of fed-batch cultivation with constant supplementation of the mixture of precursor amino acids L-histidine, L-methionine, and L-cysteine (40 g/L each) after induction of expression of *egt1* and *egt2* (Chen et al. 2022). Another *E. coli* strain harboring truncated and mutated *egt1* from *N. crassa*, mutated *egtD*, and wild-type *egtE* from *M. smegmatis* produced EGT at 5.4 g/L in 96 h of fed-batch culture (Zhang et al. 2023a). Constant feeding at 11 mL/h of precursor amino acids L-histidine, L-methionine, and L-cysteine (24 g/L each) was conducted after induction of introduced genes. A reconstructed pathway using EGT biosynthetic genes from *M. aquaticum*, omitting the *EgtA*- and *EgtC*-catalyzing reactions, enabled *E. coli* to produce EGT (Zhang et al. 2024). By deleting *metJ* and *sdaA* and overexpressing mutant genes of *serA* and *thrA*, which could enhance SAM and L-cysteine fluxes, the strain ECE14 produced EGT at 595 mg/L in 72 h of fed-batch cultivation. Superior EGT-producing *E. coli* with heterologous *egtB*, *egtD*, and *egtE* from *N. crassa* as well as manipulation of inherent biosynthetic genes for precursor amino acids was demonstrated to improve metabolic fluxes toward EGT biosynthesis in the Patent CN116121161, achieving EGT production of 7.1 g/L in 60 h of fed-batch cultivation (Wu et al. 2022). These studies suggest that not only enhancing EGT biosynthetic reactions (Fig. 2) but also modulating precursor amino acids supplies could elevate EGT production. Thus, *E. coli* strains carrying heterologous EGT biosynthetic genes are promising candidates for industrial EGT bioproduction.

*Corynebacterium glutamicum*, an actinomycete with superior amino acid bioproduction ability, has also been modified to produce EGT. By enhancing cysteine biosynthesis and introducing *egtB* from *Methylobacterium brachiatum* and *egtD* and *egtE* from *M. smegmatis*, *C. glutamicum* strains CYS- 2/pECt-Mb_egtB-Ms_egtDE produced EGT at 100 mg/L in 120 h of fed-batch cultivation in flasks using a semisynthetic medium containing 10 g/L yeast extract as a starting medium (Hirasawa et al. 2023). Another study employing *egt1* and *egt2* from *S. pombe* and balancing cysteine assimilation showed that *C. glutamicum* ET11 improved EGT yields to 264.4 mg/L in 36 h of fed-batch cultivation with starting and feeding media containing yeast extract (Kim et al. 2022).

Native EGT-producing microorganisms such as *S. pombe* (Pluskal et al. 2014), *Aspergillus oryzae* (Takusagawa et al. 2019), *M. aquaticum* (Fujitani et al. 2018), *M. neoaurum* (Xiong et al. 2022), and *C. militaris* (Chen et al. 2022) have demonstrated enhanced EGT biosynthetic ability through EGT biosynthetic gene upregulation and the improvement of precursor amino acid metabolic fluxes. Among them, *Mycolicibacterium neoaurum* strain EGT24E with overexpression of inherent EGT biosynthetic genes (*egtABCDE*) and L-histidine biosynthetic genes (*hisG* and *hisC*) as well as deletion of putative EGT-degrading enzyme (EGTase) gene produced EGT at 1.56 ± 0.27 g/L in 216 h of fed-batch cultivation with constant supplementation of the mixture of L-histidine (15 g/L), L-methionine (30 g/L), ammonium sulfate (8 g/L), and sodium thiosulfate (8 g/L) between 48 and 120 h of cultivation. Deletion of the gene encoding putative EGTase in the strain EGT24E repressed a decrease of EGT titer during fed-batch fermentation, implying the necessity of the regulation in degradation as well as synthesis for further enhancement of EGT bioproduction. A recent patent document (CN116445302) described multiple-round mutagenesis by ultraviolet irradiation and/or lithium chloride treatment with a native EGT producer *S. pombe* to improve EGT productivity (> 10 g/L of EGT production), although genomic mutations in the strain were not analyzed and no mechanistic evidence to account for such EGT productivity was presented in the document (Zhou et al. 2022). These suggest superior potential of native EGT producers for the production of EGT by genetic modification. Also, some EGT-producing strains, such as *Aspergillus* and *Pseudozyma* strains, are already utilized in industrial processes in the food and chemical industries. Although fundamental improvement in EGT biosynthesis by genetic and bioprocess engineering should be necessary, the native EGT producers reviewed here could have a great potential as host strains for industrial EGT production due to the presence of inherent biosynthetic enzymes and related metabolic processes.

Unlike model microorganisms such as *E. coli* and *S. cerevisiae*, natural EGT-producing strains lack well-established genetic engineering tools. However, among the Basidiomycetes, genetic tools have been developed for strain engineering in *Ustilago* (Olicón-Hernández et al. 2019) and *Pseudozyma* (Saika et al. 2017), leading to the creation of superior strains for enhanced glycolipid-type biosurfactant production. With these technologies, recombinant *Ustilago* and related strains may provide powerful tools for enhanced EGT production. Some *Pseudozyma* strains have already been utilized in industrial processes for production of biosurfactants (Saika et al. 2019), and process technologies for large-scale fermentation of these strains have been developed. Further improvements in productivity, as well as the development of fermentation and downstream processes, will be essential for the mass production of EGT through microbial fermentation.

## Conclusion and future perspectives

This review highlights recent progress in the microbial synthesis of EGT, along with biosynthetic pathways that may be leveraged to create novel EGT producers through synthetic biology. A wide variety of bacteria and fungi produce EGT, and significant advances have been made in elucidating its biosynthetic mechanisms. By employing these findings, useful recombinant strains for EGT production were created and multiple examples of fermentative EGT production at gram scale using the strains have been presented. Further investigation of EGT-producing microorganisms will require elucidation of the unknown physiological roles of EGT in native producers to gain a deeper understanding of its synthesis and catabolism. Additionally, the exploration of novel EGT producers may expand the available genetic tools and host strains for synthetic biology applications. By integrating these mechanistic insights into EGT biosynthesis with process engineering strategies, microbial production processes can advance to the next stage, enabling the large-scale production of EGT to meet increasing industrial demand.

## Data Availability

No datasets were generated or analysed during the current study.
